# Primary Large Mucosa-Associated Lymphoid Tissue (MALT) Lymphoma of the Gallbladder

**DOI:** 10.7759/cureus.23799

**Published:** 2022-04-04

**Authors:** Michail Papamichail, Nikolaos Dimitrokallis, Ioanna Gogoulou, Alexandros Nomikos, Christos Ioannides

**Affiliations:** 1 1st Department of Surgery and Transplantation, Evangelismos General Hospital, Athens, GRC; 2 General Surgery, Asklipieio Voulas Hospital, Athens, GRC; 3 Pathology, Asklipieio Voulas Hospital, Athens, GRC

**Keywords:** mucosa-associated lymphoid tissue, gallbladder tumor, primary lymphoma, lymphoma, malt, gallbladder

## Abstract

Primary lymphoma of the gallbladder (GB) is a rare condition, and very few cases have been reported so far. Diagnosis is usually made after surgery of suspicious GB mass, which is often difficult to differentiate from GB carcinoma. The GB wall does not contain lymphoid tissue, and tumors arise at the submucosal layer. Stone disease and chronic inflammation may contribute to its pathogenesis. Treatment consists of surgical resection followed by adjuvant therapy in selected cases. We present a case of an unusual, large-sized mucosa-associated lymphoid tissue (MALT) lymphoma of the GB.

## Introduction

Gallbladder (GB) lymphoma accounts for less than 0.2% of all GB malignancies, and very few cases have been reported so far. It can be either primary, in the form of extra-nodal non-Hodgkin lymphoma solely originating in the GB, or secondary (more common), as a result of a general lymphoproliferative disease with or without multiple other sites involved within the gastrointestinal (GI) tract [1]. The most common subtypes are mucosa-associated lymphoid tissue (MALT) (40%) and diffuse large B-cell lymphoma (DLBCL) [1,2]. MALT lymphoma of the GB appears to be more frequent in females (65%), with patient age ranging from 31 to 84 years [3].

Pathogenesis is not clear as the GB wall lacks lymphoid tissue, but it is hypothesized that chronic irritation and possible bacterial translocation in the background of lithiasis may cause lymphocyte migration and infiltration. In an alternative pathway, an antigenic stimulation causes chromosomal translocation resulting in the synthesis of a fusion protein (API2-MALT1) that inhibits apoptosis and causes antigen-independent proliferation [4,5].

GB lymphoma has an indolent course and usually does not give systemic symptoms, but most commonly patients report nonspecific upper abdominal symptoms (right upper quadrant pain, nausea vomiting, etc.) possibly due to concomitant lithalsas and chronic inflammation. However, stone disease was found only in 65% of cases in one study [3]. The tumor size varies from a few millimeters to 3-4 cm and is confined to the submucosal layer without penetrating the mucosa of the GB.

We present a case of a primary GB MALT lymphoma of an unusually large size.

## Case presentation

**Figure 1:**
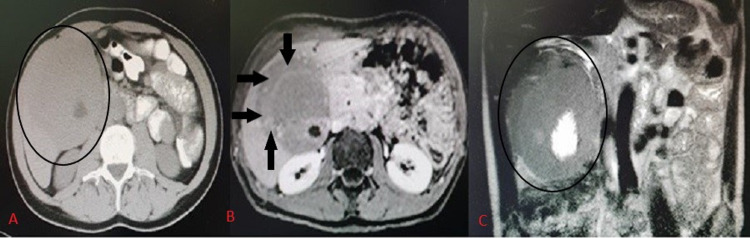
A 15-cm soft GB mass extending to the central liver parenchyma and hepatic hilum without intrahepatic duct dilatation. The big GB mass is included in circles on noncontrast enhanced CT (A) and MRI (C). Extension in liver parenchyma is shown with arrows in MRI of the liver (B). GB, gallbladder

A 39-year-old male with a two-month history of low-grade fever, weakness, fatigue, and a weight loss of 5 kilograms was referred to our unit by his general practitioner after he was found to have a palpable right upper quadrant mass. Imaging revealed a 15-cm soft GB mass extending to the central liver parenchyma and hepatic hilum (Figure 1). There were no abnormal liver function tests or any intrahepatic duct dilatation. Tumor markers were normal. Upper and lower GI endoscopies did not reveal any abnormalities. Due to atypical imaging features, an exploratory laparotomy was offered.

**Figure 2:**
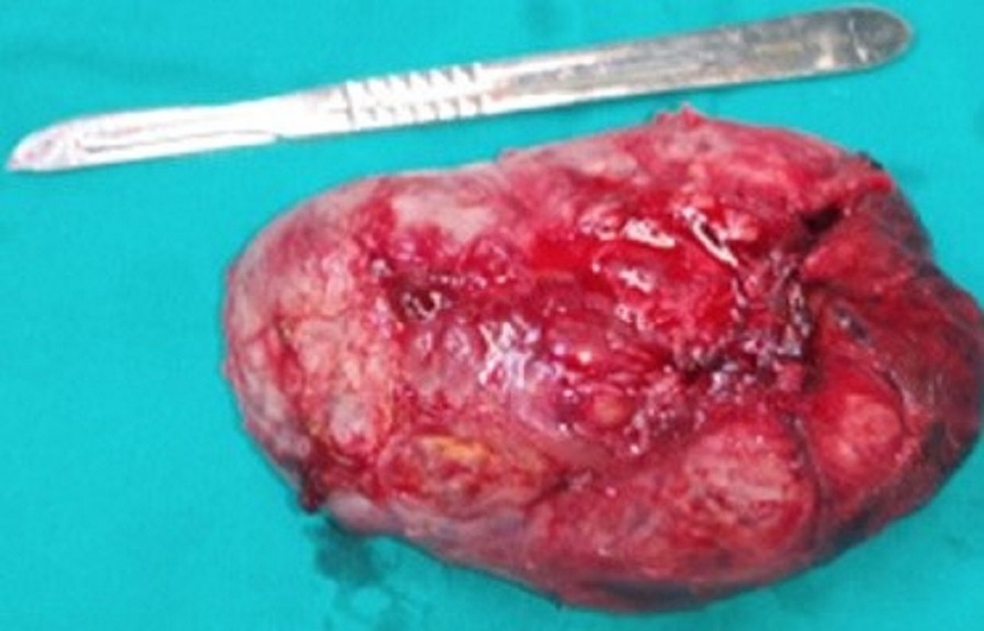
Cholecystectomy specimen with en block neoplastic tissue from the hepatoduodenal ligament.

The patient underwent an open resection of the GB mass without liver or bile duct resection. Intraoperatively, a soft mass originating from the GB involved the entirety of it pressing on the liver parenchyma and extended distally to the hepatoduodenal ligament occupying the entire hilum. The macroscopic appearance was not typical of adenocarcinoma, and a frozen section was from the GB wall that came back positive for lymphoepithelial neoplasia. Following this result, the decision was made to not proceed to a radical cholecystectomy but rather perform a simple cholecystectomy removing the neoplastic tissue. The mass despite its adherence was peeled carefully from the liver bed, and a cholecystectomy was performed. Additional neoplastic tissue from the hepatoduodenal ligament was peeled and removed en block with the GB specimen (Figure 2).

**Figure 3:**
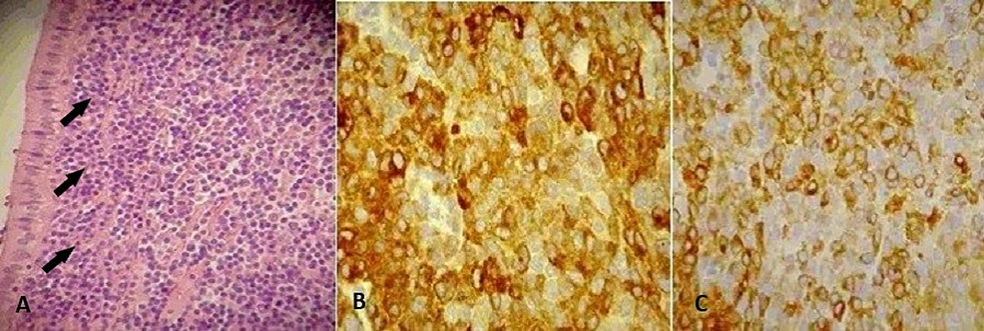
Microscopic examination findings. (A) Hematoxylin and eosin x100:  neoplastic cells infiltrate the lamina propria in a diffuse pattern (black arrows). (B) CD 20 x100: tumor cells express CD20. (C) CD79a x200: tumor cells express CD79a.

Microscopic examination revealed a dense lymphoid population composed of medium-sized cells with round to slightly irregular nuclei, inconspicuous nucleoli, and a small amount of cytoplasm, arranged diffusely within all layers of the GB wall and focally the mucosa. The lymphoid cells were not breaking the serosa or the connective tissue toward the liver. At the immunohistochemical examination performed, the tumor cells were strongly positive for CD20, CD79a, and BCL-2, and negative for CD3, CD10, CD5, and CD23 (Figure 3). The histological diagnosis of MALT lymphoma was confirmed. The patient had an uneventful recovery and was discharged four days post-operatively. He received six cycles of adjuvant rituximab-bendamustine followed by another six cycles of maintenance rituximab. The patient remains disease-free two years after the surgery.

## Discussion

GB lymphomas reflect on the lymphomas observed in the GI tract, although the incidence is less frequent than in other sites (e.g., stomach). The most common histological subtypes are MALT, DLBCL, and follicular lymphoma [6]. In one study including 56,000 autopsies, histological examination revealed 216 cases of carcinoma but no cases of lymphoma [7]. In two other studies of 36,355 and 1,452 cholecystectomies, only three cases and one case, respectively, of GB lymphoma were incidentally discovered [1,8]. The tumor size and appearance are not typical. Submucosal thickening with few atypical cells or a small (0.5-4 cm) tumor with discrete boundaries or a polypoid transformation with neoplastic lymphoproliferative cells have all been described [9-11]. We first reported an unusual appearance of a bulky lymphoid tumor (14 cm) attached to the liver bed and hilum.

Symptoms are insufficient to direct diagnosis as they are infrequent and non-specific, and if existing, they are usually related to concomitant stone disease [12]. Imaging may reveal abnormal thickening of the GB wall, or if a tumor is revealed, features are indistinguishable from other types of neoplasm (e.g., adenocarcinoma) [12]. Findings such as a hypoechoic homogeneous submucosal lesion with a smooth surface in ultrasonography or lesions with low-signal intensity on the T1-weighted sequences and high-signal intensity on T2-weighted sequences compared with liver parenchyma on MRI may raise suspicion. DLBCLs tend to form a solid and bulky mass with irregular wall thickening as opposed to MALT or follicular type where thickening of the GB is less prominent [13]. Histological examination will provide definitive diagnosis [3]. In our case, the large size and the pattern of local spread within the liver hilum and GB bed were unusual for a primary MALT lymphoma.

The majority of cases reported were discovered incidentally, but if a tumor is suspected on imaging, management should be similar as in GB adenocarcinoma. Open or minimal invasive cholecystectomy with possible GB bed liver resection based on the frozen section of the GB and cystic duct stump is recommended. It has been shown that at least four lymph nodes are required for staging purposes, with six being the number advised by the American Joint Committee on Cancer [14]. Initial diagnostic laparoscopy is optional but usually cannot differentiate lymphoma from carcinoma. Likewise, lymph node sampling for frozen section before cholecystectomy is unlikely to be efficient in providing accurate diagnosis, and negative for malignancy results should not impact decision for cholecystectomy [12]. In our case, initial frozen section was helpful in excluding GB carcinoma; therefore, a less radical resection was proposed, and due to the soft consistency of the tumor, it was possible to peel this off from the liver bed and the hilar elements successfully. We did not achieve the six lymph nodes suggested by the American Joint Committee on Cancer; however, given the nature of the tumor, we opted to not re-explore our patient for the sole purposes of lymph node harvesting.

Due to the very small number of cases and the fact that confirmation of diagnosis usually follows surgery, a treatment protocol is difficult to establish. Factors that can influence the decision for adjuvant treatment are the presence of distant disease, histological subtype, incomplete surgical margins, secondary GB lymphoma originating from another site within the GI tract, bone marrow biopsy results, and age and fitness of the patient. In general, simple R0 cholecystectomy appears sufficient in the majority of cases. In our case and in the light of a less radical resection, we offered chemotherapy, as described previously. Our patient responded very well and remains free of disease two years after resection.

## Conclusions

Primary GB MALT lymphomas are particularly rare, and imaging findings are nondiagnostic. Tissue diagnosis is possible with intraoperative frozen section examination. Due to the soft consistency of the tumor, it is possible to achieve a complete resection without necessarily proceeding to liver bed resection at the time of surgery. With the combination of a complete resection and adjuvant chemotherapy, our patient has so far remained free of disease two years after surgery.
